# Online learning for continuous professional development of healthcare workers: an exploratory study on perceptions of healthcare managers in Rwanda

**DOI:** 10.1186/s12909-022-03938-y

**Published:** 2022-12-08

**Authors:** Jean Claude Byungura, Gerard Nyiringango, Uno Fors, Elenita Forsberg, David K. Tumusiime

**Affiliations:** 1 Department of Business Information Technology, University of Rwanda, Kigali, Rwanda; 2 Department of Computer and Systems Sciences, Stockholm University, Stockholm, Sweden; 3 Department of Nursing, University of Rwanda, Kigali, Rwanda; 4 School of Health and Welfare, Halmstad University, Halmstad, Sweden; 5 Center of Excellence in Biomedical Engineering and E-health, University of Rwanda, Kigali, Rwanda

**Keywords:** Online learning, Continuous professional development, Health professionals, Digital health, Healthcare managers

## Abstract

**Background:**

Due to outbreaks of new diseases, development of new treatment regimens and requirement of evidence-based practice, health professionals continuously need to acquire updated knowledge and skills. This type of learning is known as continuous professional development (CPD). The scarcity of skilled health care professionals in developing countries further increases the need of CPD. Traditionally, face-to-face approach has been preferred as the best mode of CPD. Currently, health professionals have started using online learning for continued professional growth in different parts of the world. Consequently, research studies from different settings are needed to investigate the significance of online learning for CPD. Therefore, the aim of this study was to investigate the importance and challenges attributed to online learning by the managers of health facilities in Rwanda. Moreover, the study aimed to identify the status of infrastructures that could support online CPD, and assess the perceived enhancement and barriers for implementing online CPD.

**Methods:**

The study used a convergence mixed-method design to explore quantitative and qualitative data from 42 health care managers. A descriptive analysis was conducted on quantitative data while qualitative data were thematically analyzed to inform the study findings.

**Results:**

It was revealed that 90.5% of managers, who participated in this study, consider positively the use of online learning for CPD. All managers acknowledged that online learning could improve the knowledge and practice skills of health care professionals. Nevertheless, 52.4% of health institutions who participated in this study currently do not use online for CPD. Participants demonstrated challenges such as the lack of access to digital devices, poor or lack of internet access, poor online learning design, low digital skills of healthcare professionals, lack of time dedicated to online learning, and heavy workload of staff.

**Conclusion:**

These findings indicate then that the managers of health institutions value the importance of online learning for CPD of health professionals. However, online learning should be designed to fit for the purpose and with a high consideration on needs and preferences of healthcare professionals and thereby improve information communication technology infrastructure that support online learning for CPD. Traditional in-person CPD courses are still recommended in health institutions with shortage in resources and technology. Also, the barriers of online CPD delivery such as low internet connectivity and lack of access to digital devices by healthcare professionals need to be co-creatively addressed through the pyramidal structure of the Rwandan health system.

## Background

Globally, there seems to be an increasing deficit of well-trained healthcare professionals (HCPs) in most countries in the world [1, 2] which entail that the health institutions aspire to train more pre-service as well as in-service human capital by all the available means to address this challenge. In 2014, a shortage of 2.4 million HCPs (mainly doctors and nurses) was reported by the World Health Organization (WHO), mainly in Asia and Sub-Saharan Africa. This trend of HCP shortage is still apparent in several countries across the globe including Rwanda [2] where it was reported in 2018 by WHO that there is only 1.3 doctors and 12 nurses/midwives per 10,000 inhabitants [3]. Thus, without an ample number of well-trained HCPs and supported for effective continuous professional development (CPD), there is a high risk of failing to achieve the health-related Sustainable Development Goal 3 (Good Health and Wellbeing).

There is, therefore, a clear need for in-service healthcare professionals to continuously update their knowledge and skills so that they can stay dynamic and confident in managing the increasing and changing health challenges. The knowledge acquired via basic medical education sometimes gets obsolete, and thus a strategic approach for regular updates needs to be established. In this context, ministries of health from several countries strategize the provision and access of life-long learning, commonly known as continuing professional development (CPD) [4, 5]. This strategy ensures that healthcare professionals (HCPs) can update their knowledge and practical skills during various training events. Current information technology solutions are also seen as enablers for this strategy for online CPD delivery [2, 6], and HCPs are encouraged to use information technologies to update their specialties at the workplace [7, 8]. Consequently, online CPD programs are being adopted in both developed and low-resource countries [9–11]. Despite the undisputable role of emerging technologies in support for modernizing CPD delivery in healthcare sector, some CPD courses may require face-to-face lectures or practical workshops or seminars. For example, the CPD courses that aim at developing new practical hands-on skills such as surgical practices or the usage of new laboratory equipment need to be delivered in-person. However, with the rise of the COVID-19 pandemic, online learning systems are seen as a strategic digital solution to ensure CPD delivery to in-service healthcare professionals from across the world, despite some contextual settings, types of CPD courses and related challenges [12, 13].

Once online CPD technology has been well-integrated and provides flexible and self-paced learning capabilities adapted to the local contexts, it provides opportunities for in-service healthcare providers to acquire and update knowledge, skills, and attitudes in their field [9]. Studies indicate the usefulness of online learning for CPD in the health sector to develop innovative knowledge, skills, and attitudes of health workers and keep them up to date to improve service delivery [2, 9, 13]. In some contexts, for example, where there is a shortage of health workers, online learning has been adopted to update skills and knowledge through CPD [7, 14, 15] for health workers such as physicians, nurses, and midwives without leaving the workplace. Similarly, an enabling online learning environment for in-service HCPs helps them build self-confidence in clinical reasoning and decision-making on patients’ cases.

On the other hand, from an instructional perspective, it has been argued [2, 5] that online CPD courses need to be well-designed considering health workers’ needs and also based on sound learning theories. From a technological perspective, one study proposes that online CPD courses must be interactive, easy to access, affordable, and the participants should be facilitated to access digital devices and acquire basic digital skills [2]. In addition, the literature reports different challenges of online learning for medical education, particularly in low and middle-income countries, including the language of instruction [16] and lack of adequate ICT infrastructure [9, 17, 18]. This mixture of benefits and challenges requires healthcare managers’ coordination to create an enabling online learning environment that can support online CPD. The situation calls for strategic alignment of online learning systems, needs for CPD, and institutional involvement in the entire process as a critical phase to ensure effective life-long learning of in-service HCPs. In Rwanda, for example, HCPs at each level are required to undertake continuous in-service training as a mandatory and strategic requirement by the professional regulatory boards [4]. This mandatory CPD helps HCPs maintain their skills and competencies needed to deliver effective and high-quality healthcare service, but the policy is silent regarding the mode of delivery.

In recent years, the Human Resource for Health (HRH) program boosted in-service training. While the mission of health staff in HRH program was to ensure support in training of pre-service health professional, their presence in clinical placements and their close collaboration with healthcare managers benefited CPD of in-service healthcare providers including physicians, nurses, midwives, and oral healthcare providers. This face-to-face CPD benefited healthcare providers at referral and district hospitals and it might indirectly have benefited nurses and midwives at health centers. According to the Rwandan health labor market analysis report (2019), the total number of registered health professionals was 16,698. These include nurses and midwives (15,050) and (1,648) medical doctors [19]. These healthcare workers operate within a resource constrained health system with the health workforce shortage, which therefore entails the active role of healthcare managers. The later occupy managerial positions at the Ministry of Health, Referral Hospitals and District Hospitals. The country also registers 499 health centers that are also headed by a healthcare manager who oversees and coordinates the health center activities including the advocacy of its staff for undertaking CPD and get their licenses renewals.

In the Rwandan health system, nurses and midwives are responsible of patient care at health centers and health posts and most of these health centers are led by nurses. Nurses and midwives at the level of health center are in charge of diagnosis, treatment, and if necessary, transfer the patient to an advanced level according to the health system structure. While this approach addresses the challenge of physician shortage in Rwanda, it may come with its challenges. Nurses and midwives who are initially trained to manage health cases related to nursing and midwifery profession they get overstretched to acquire vast knowledge and skills for diagnosing and managing or transferring complicated cases at their level. This contextual situation re-affirms the need of CPD at this level of health centers and more importantly those computer-based CPDs that can be attended while at work, with the help of online learning systems. In this regard, the role of healthcare managers is highly significant in HRH initiatives across the national health system structure for effective CPD implementation. Also, the way healthcare managers perceive and can actually implement strategies and approaches to online learning systems, as a means of effective CPD delivery within their contextual settings, is paramount.

Hence, the effectiveness of online learning for CPD is conditioned by several factors, including, for example, the healthcare managers’ level of understanding of the potential role of online learning for CPD, the strategy in place, the instructional infrastructure, and the level of awareness among healthcare managers as well as other healthcare professionals. Therefore, a supporting organization of approach, people, and infrastructure were also reported as essential for effective online learning for HCPs and facilitating the online learning for CPD [20]. Effective online learning for CPD and formal health education require that stakeholders are highly involved in the planning and supporting these initiatives related to online learning uptake [21].

As the drivers of this move, healthcare managers must understand the importance of online CPD for in-service healthcare workers to better plan how the latter can be supported at the institutional level. Healthcare managers are one of the most important stakeholder groups that should develop an environment conducive to HCPs adopting online learning for CPD through establishing clear related policies and other institutional enablers [13]. Their level of understanding of their institutional online learning environment helps in developing and owning a related strategy for supporting online CPD pertaining courses. However, it is still unclear in the literature how managers from the healthcare sector understand and interpret the trends in online learning for CPDs and strategize this trend at the institutional level. The available literature on online CPD for HCPs and practical skills has mostly focused on practitioners’ perspectives [11, 22, 23], with others emphasizing the importance of understanding tutors’ and trainers’ opinions towards online learning for health education [7, 21] and medical students [6, 13]. More particularly, research on online CPD on the Rwandan context has followed the same trend of emphasizing medical students’ and teachers’ stands towards online learning [24–27].

Consequently, the current status of online learning adoption for CPD and the possible associated challenges based on the perspectives of health managers in Rwanda are under-researched. Therefore, the present study focuses on this purpose by exploring the perspectives of healthcare managers in terms of importance attributed to online CPD, barriers, and enhancing factors for improving the CPD of in-service healthcare professionals in Rwanda. This explorative study specifically aims to:

- Investigate the importance and associated aspects of online learning for CPDs.
- Establish the status of digital infrastructure that supports online learning for CPDs.
- Identify the perceived enhancing factors for health professionals’ online learning adoption for CPD.
- Ascertain the barriers of online learning adoption for CPD from healthcare managers’ perspectives.

Most clinical professions need CPD due to the fast and innovative development of almost all healthcare areas. However, the specific CPD need and thus requirements for a general practicing physician, a specialist physician, a nurse or a physiotherapist may vary considerably. However, this study was focusing on investigating the possibilities to use computer-based CPD as a delivery method, regardless of the specific content, which we believe is applicable more or less for any healthcare profession.

The present study is significant as the results have the potential to inform the future of CPD planning and delivery considering the intervention of emerging technologies. Potential insights into possible training requirements and improvement of potential enhancing factors to adopting online learning for CPD were also expected to be reported in this study. Accordingly, this study may also guide further development of online learning for CPD in local contexts such as health centers and district hospitals that aspire to develop health workers’ innovative skills, knowledge, and self-confidence in clinical practice.

## Methods

### Study area

We collected empirical data regarding online learning for supporting CPD programs from the managers with leadership responsibilities at 31 health centers, 9 district hospitals, and 2 referral hospitals in Rwanda. Participating institutions in this study are located in both rural, urban and semi-urban areas and all the four provinces of Rwanda as well as the main capital of Kigali City were represented. The geo-location of health facilities data including hospitals and health centers were acquired from Rwanda Biomedical Centre.

### Study design

As an exploratory study, we applied a convergence mixed methods with both quantitative and qualitative approaches to data collection, analysis and interpretation of results [28]. The rationale for applying a mixed method approach in this study was to enable researchers get more in-depth data and findings that can help in achieving different study objectives. In addition, this triangulation design approach was applied to ensure the corrected data using traditional surveys can be complimented by the in-depth interview data [28, 29] collected from the healthcare managers. The survey design helps in providing numerical descriptive data about trends, opinions, perceptions or attitudes from a sample of the study population [28]. With the mixed methods design, the survey research was applied to collect quantitative data related to the importance of online learning for CPD as well as the status of internet connection at the healthcare institutions. These data are the opinions and perceptions of the healthcare staff occupying the managerial roles. On the other side, the mixed method study design facilitated the collection of qualitative data about the perceived challenges to the adoption of online learning for CPD. A stratified purposive sampling technique [28, 30] was used to identify representative institutions to select respondents (*n = 56*). Potential respondents to the survey questionnaire for this study comprise physicians, nurses, midwives and others with managerial positions from health centers, district, and referral hospitals. The targeted institutions included 40 health centers and 10 district hospitals with a mixture of urban, semi-urban and rural areas.

**Fig. 1:**
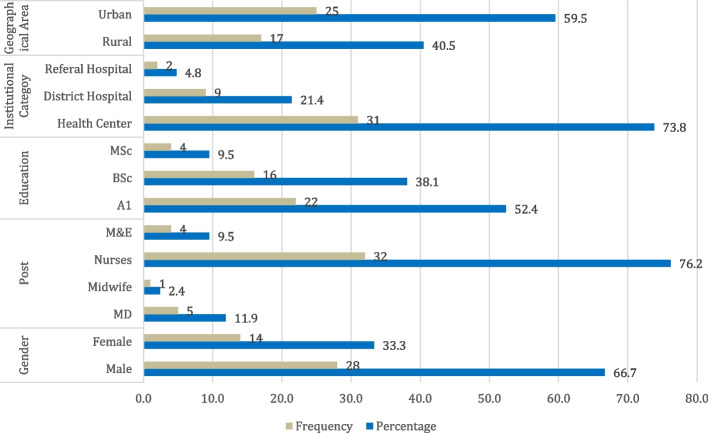
Respondents’ demographic information

In addition, two referral hospitals (one from urban and one from rural area) were included, resulting in a sample of 52 institutions totally. Due to unavailability of managers at some institutions, we managed to collect data from 42 participants, representing a survey response rate of 75%. A detailed background of respondents is visualized in Fig. 1. As we aimed to understand different perspectives of online learning for CPD programs, with regard to the geographical location, the sample selection considered both rural and urban healthcare managers.

### Data collection

After obtaining a research ethical clearance, researchers collected data during the period of May to October 2021 using questionnaire and interviews as research instruments. Data were purposively collected using internet-based cross-sectional survey questionnaire [28] which was designed with both open and close-ended questions. The designed survey questionnaire includes a combination of Yes/No/Don’t know types, tick-type variables and five-point Likert scale questions. This data collection instrument was preferred because it is easy to design and it helps for rapid turnaround in data collection [28]. Additionally, an interview guide was designed as well to support further data acquisition, in line with the study objectives to collect in-depth qualitative data that provide the perspectives of health institution’s managers in line with the needs, challenges and enhancing factors for online learning for CPD programs. Semi-structured interviews were conducted with selected 14 healthcare managers from district hospitals as well as the health centers.

To ensure the study reliability and validity using a mixed method design [28, 29], both the survey questionnaire and the interview guide were designed collaboratively by the research team and among them include experts in health informatics, nursing, public health, medical education, and E-learning systems. Considering that this study is exploratory, researchers were guided by an inductive reasoning and thus a survey tool was self-developed based on prior experiences and specific observations by the researchers. However, even if the survey is, to a certain degree, based on similar surveys used in previous research projects, the concurrent validity [28] was assessed before administering the survey instrument to the respondents. An online version of the final survey instrument was designed using Google form and a link was made available for testing. Prior to administering the online survey questionnaire, it was reviewed by other staff at the department of nursing (who are familiar with CPD programs) and the E-learning specialists from the department of business information technology at University of Rwanda. Thereafter, the online survey questionnaire version was piloted with the office in charge of E-learning and CPD at the Ministry of Health of Rwanda and to some faculty staff to ensure its content validity and concurrent validity respectively. Seven respondents participated in the pre-testing of the online survey instrument as well as the review of the interview guide.

Based on the provided suggestions, further revisions were made to the survey questionnaire to ensure its clarity, structure as well as the format of some wording, questions and variables. Based on provided suggestion, further revisions were made to the survey questionnaire to increase its clarity, structure as well as the format of some wording, questions and variables.

### Data analysis

Quantitative data, collected from survey questionnaires, were imported into and analyzed using SPSS version 23 and Microsoft Excel for data visualization. The analysis produced basic descriptive statistics such as frequencies and percentage that were generated from respondents’ responses. The analyzed responses were categorized and summaries were visualized in tables and figures using Microsoft Excel package and then shared among the research team to ensure neutrality and alignment with the findings and the study objectives.

Qualitative data include the interviews and the responses to the open-ended questions from the survey. Using Microsoft Excel, a simplified grounded theory approach [31] was applied to code the follow up responses collected from the online survey. The recorded interviews were also transcribed verbatim by the authors. With the verbatim transcription process, the recorded data have been texted exactly the way they were said by respondents [32]. The final interview transcripts were imported into MAXQDA, a qualitative data analysis software [33] for further analysis. This computer-based data analysis tool was used to visualize the barriers and enhancing factors of online learning for CPD programs using the interview manuscripts. A thematic analysis approach [31, 32] was therefore applied, following an inductive reasoning to ascertain the underlying themes which were later categorized into predefined codes. This approach was also important to generate the meaning attributed to the needs, importance and challenges and enhancing factors for online learning for CPD as expressed by respondents.

### Ethical consideration

Prior to engaging in the data collection, ethical issues were though about with regard to the respondents’ informed consent, their confidentiality, anonymity and their willingness to participate in the study [28, 34].

The study was ethically approved by the University of Rwanda College of Medicine and Health Sciences’ Institutional Review Board (IRB No. 240/CMHS IRB/2020) and endorsed by Rwanda National Ethics Committee (Review Approval Notice: No.156/RNEC/2021). In addition, the study was also approved scientifically by National Health Research Committee (NHRC) with reference number: NHRC/2020/PROT/027).

The ethical approval notices were presented to the heads of the visited institutions (the main respondents for this study) as evidence that this study comply with the ethical clearance in Rwanda. and when an agreement is reached, researchers were directed to the potential respondents.

Prior to administering the survey questionnaire link and setting up the interviews, respondents were informed about the purpose of the research and that the data will only be used for this research. Informed consents were provided by the respondents and it was clearly informed that the participation in the study was voluntary and each one was free to withdraw from the study at any time. In addition, confidentiality and anonymity were also assured [28] during the collection and analysis of data as well as reporting the study results by following the key principles of research ethics of not disclosing the identity of respondents.

## Results

### Respondents background

Among the 56 respondents to which the link to the online questionnaire was administered, 42 health managers successfully completed it. The entirely completed survey questionnaires give a response rate of (75%). To avoid bias in data analysis, incomplete survey questionnaires were not considered in this study.

As it can be observed from Fig. 1, health managers who participated in this study (66.7%) are male. The findings also indicate that the majority of respondents are nurses mainly heading the health centers (76.2%) while (11.9%) are medical doctors representing district or referral hospitals. Some health institutions were represented by monitoring and evaluation specialists (9.5%). Of the total respondents, (40.5%) are from rural area institutions while (59.5%) are based in institutions from urban area.

### Importance of online learning for CPD

The first goal of this study was to investigate the extent to which online learning for CPD is important based on healthcare manager’s perception.

The findings, indicate a high-level importance that healthcare managers attribute to the online learning for CPD of in-service healthcare professionals. This study revealed that 100% of the total respondents find online learning as very (90.5%) or rather (9.5%) important option to train in-service healthcare workers at the workplace.

**Table 1:** Aspects of Online Learning importance for CPD delivery

		Freq (%)
Considering the needs for improving employee’s knowledge, attitude and practice	Yes	42 (100)
	No	0 (0)
	Don’t Know	0 (0)
Current use of Online Learning for CPD	Yes	19 ((45.2)
	No	22 (52.4)
	Don’t Know	0 (0)
Online Learning: Strategy for CPD delivery	Yes	35 (83.3)
	No	7 (16.8)
	Don’t Know	0 (0)
Online Learning: Strategy to improve quality of healthcare delivery	Yes	36 (85.7)
	No	2 (4.8)
	Don’t Know	4 (9.5)
Research: Online Learning feasibility for CPD delivery	Yes	12 (28.6)
	No	27 (64.3)
	Don’t Know	3 (7.1)
Need Analysis: Online Learning for CPD delivery	Yes	18 (42.9)
	No	21 (50)
	Don’t Know	3 (7.1)
Alignment of Online learning strategy with the institution’s mission	Strongly agree	16 (38.1)
	Agree	19 (45.2)
	Neutral	4 (9.5)
	Disagree	3 (7.1)
	Strongly Disagree	0 (0)

The findings presented in Table 1 indicate that all health managers (100%) that participated in this study agree that their institutions consider highly the need for improving employees’ knowledge, attitude and innovative practice of healthcare delivery through online learning. In addition, the majority of respondents reported that their institutions consider online learning as a strategy to improve CPD delivery (83.3%) as well as quality of healthcare delivery (85.7%). However, there are still several health institutions (52.4%) that currently do not use online learning platforms for CPD.

**Fig. 2:**
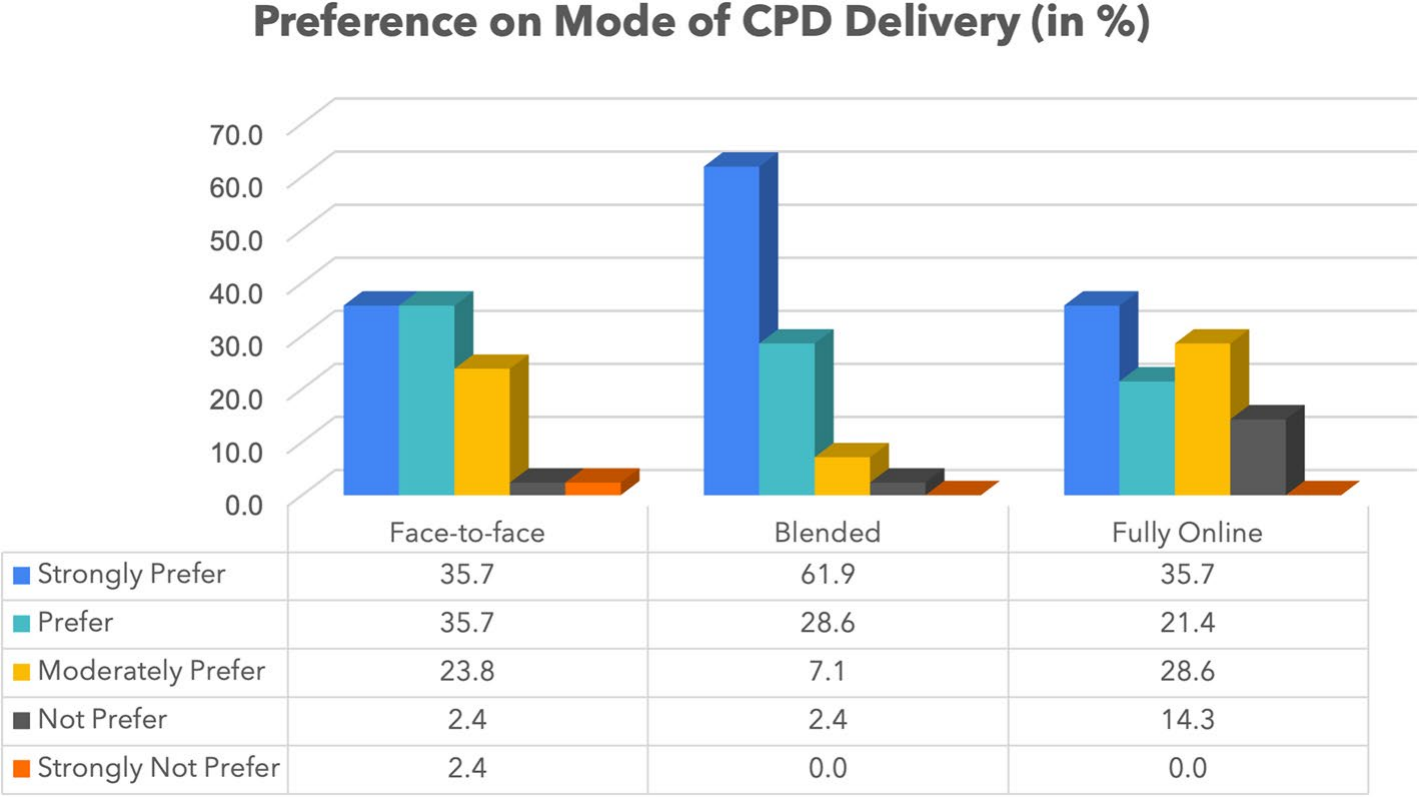
Preferences on Modes of CPD delivery

As it can be observed from Fig. 2, the findings indicate that several health managers (90.5%) prefer blended learning mode for CPD delivery while (71.4%) opt for face-to-face learning mode and only (51.1%) prefer CPD that is organized fully online.

### Readiness of online learning for CPD

**Fig. 3:**
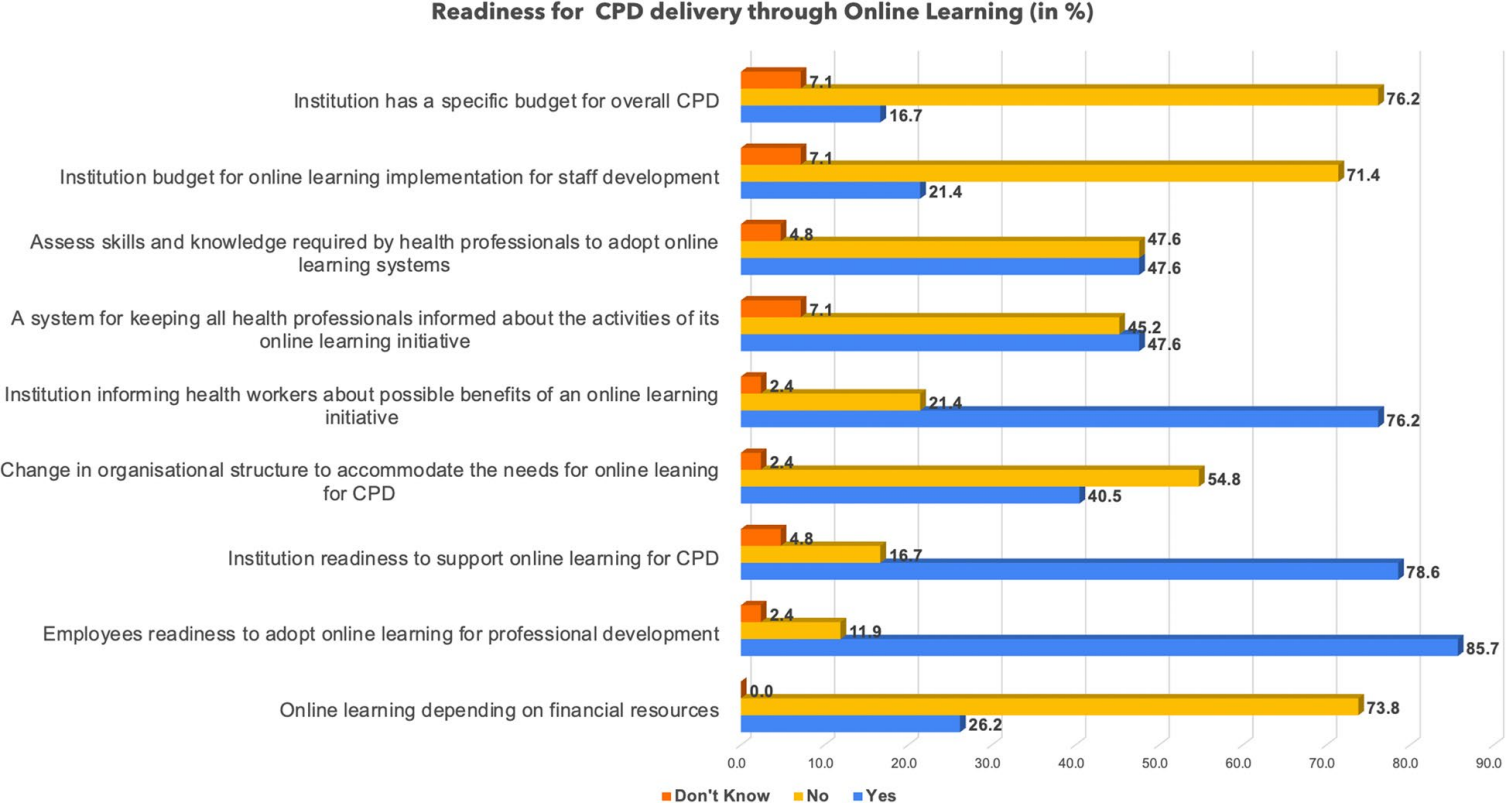
Institutional readiness to adopt online learning for CPD delivery

In this study, authors were also interested in enlightening health managers’ perceptions about the level of readiness on some aspects that are aligned with the adoption of online learning for CPD by health professionals. The related findings are reported in Fig. 3 below.

Figure 3 visualizes the findings about the readiness of online learning at various health institutions. The managers estimate (85.7%) of employees are ready for online CPD. In addition, they estimate that institutions are ready to integrate online learning systems to support CPD delivery by (78.6%). The same findings enlighten that the majority of health institutions inform regularly their staff about the benefits of online learning initiatives (76.2%).

On the other hand, other aspects of readiness such as the institution having a specific budget for CPD or for supporting staff’s online learning, and the change in organizational structure to accommodate online learning for CPD registered a low level of readiness.

### Perceptions on digital infrastructure in place

**Fig. 4:**
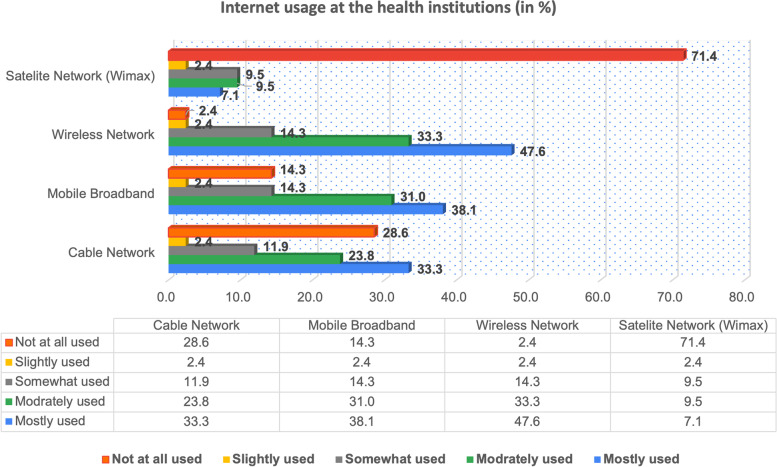
Usage of Internet at the health institutions

For health professionals to adopt online learning through their CPD, a reliable infrastructure such as internet connectivity and devices have to be in place. Therefore, we explored the perceptions of health managers regarding the usage of the four types of internet access by in-service health professionals. The related findings are summarized in Fig. 4 below.

As visualized in Fig. 4, among the four types of internet connections, health managers reported that Wireless Network is mostly used (47.6%) by healthcare professionals followed by Mobile Broadband (38.1%).

**Fig. 5:**
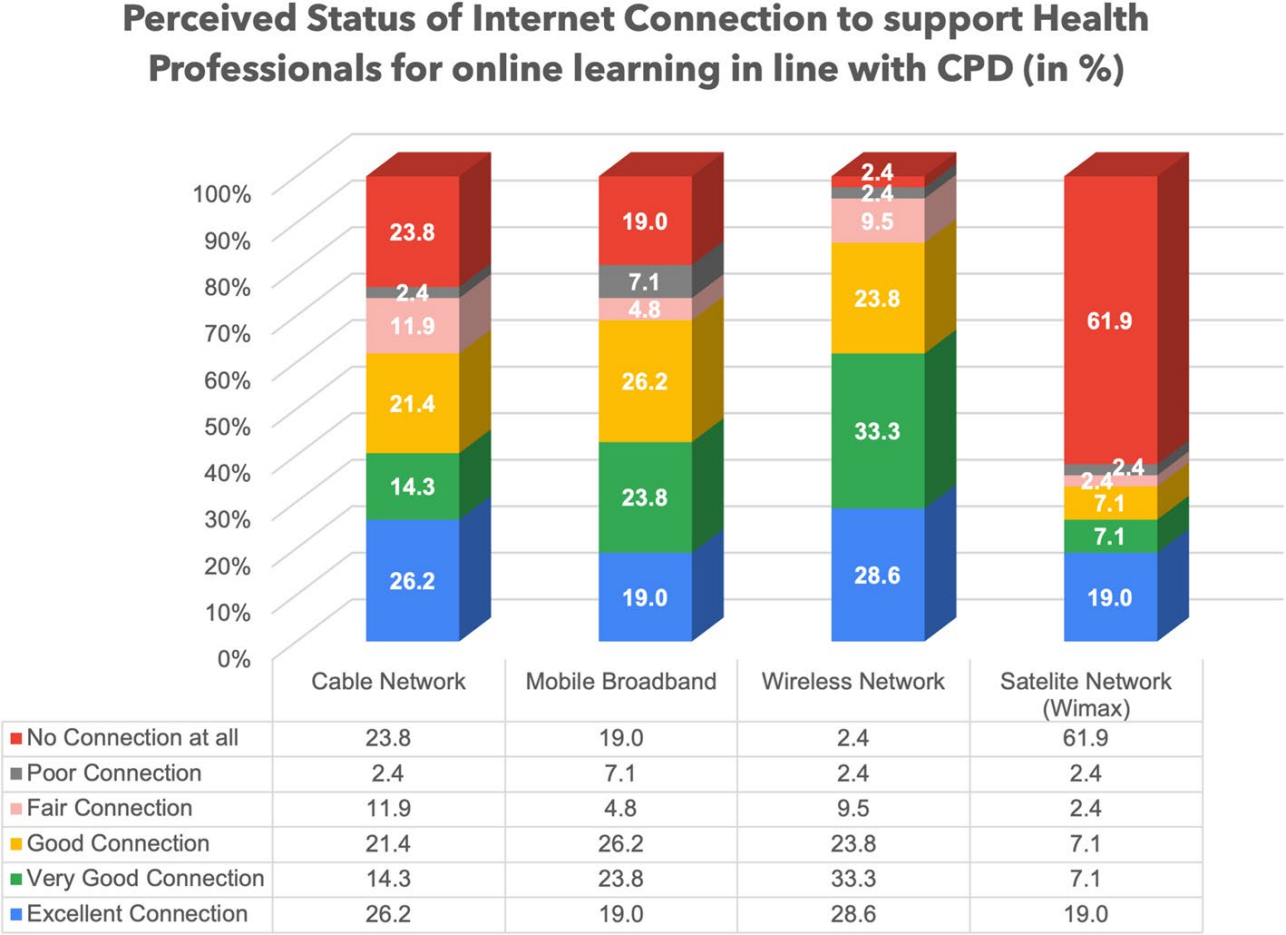
Status of Internet Connection at the health institutions

In as far as the usage of internet for online learning for CPD is concerned, we were also interested in understanding the status/level of each internet connection being used to support health professionals at the workplace. The findings related to this internet connection status, as reported by health managers, are also visualized in Fig. 5 below.

Regarding the status of current internet connection as presented in Fig. 5, respondents perceive Wireless Connection as excellent and good by (85.7%) followed by Mobile Broadband Connection (69.1%). The Cable (wired) Network was also reported to be on average good and reliable in some health institutions (61.9%).

### Perceived factors enhancing staff online learning for CPD

**Fig. 6:**
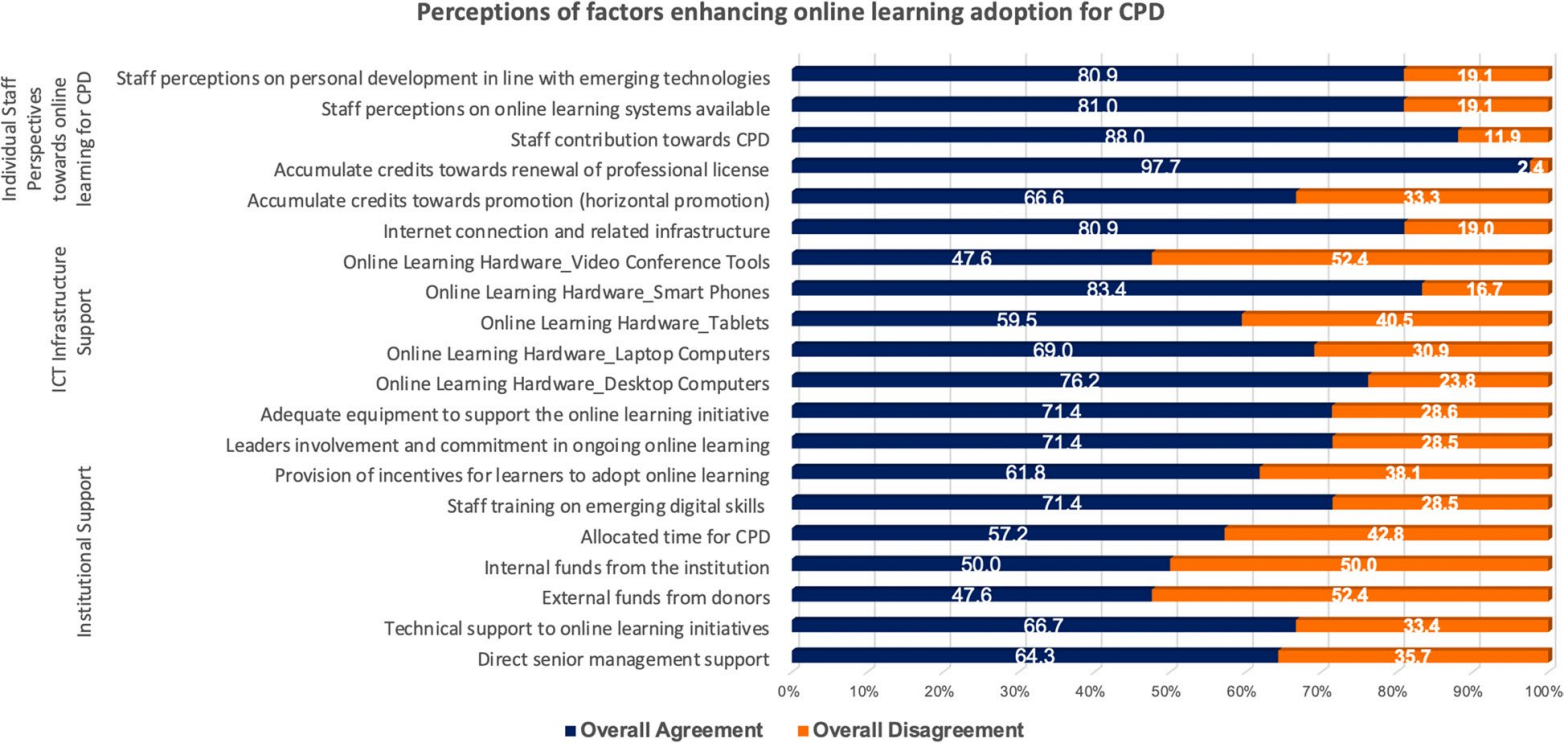
Perceptions on contributing factors to the adoption of online learning for CPD

In this study we explored the perceptions of respondents regarding the factors that could trigger in-service health professional’s adoption of online learning in their CPD programs. Related findings are reported in Fig. 6 below.

As it can be observed from Fig. 6, the proposed enhancing factors of online learning adoption for CPD were clustered into three categories namely, (1) *Individual Staff perspectives towards online learning for CPD*, (2) *ICT infrastructure support* and (3) *Institutional Support.* The findings presented under these clusters indicate that the accumulation of credits for the renewal of professional license (97.7%) was perceived as the most important factor that could enhance the adoption of online learning for CPD. Accordingly, the health managers consider highly the staff contribution towards CPD (88%), staff perceptions towards the available online learning systems (81%) as well as their personal development in line with emerging technologies (80.9%) as the key factors to enhance the adoption of online learning for CPD.

Accordingly, under the ICT infrastructure support category, health managers consider mainly the ownership of smartphones (88.3%), a good internet connection (80.9%) and the availability of enough desktop computers (76.2%) as the key factors to enhance online learning for CPD. Other factors proposed under the institutional support cluster recorded also some positive rate of 50%, which entail that they are not to ignore when institutions are planning of online learning environment to support CPD. Overall, these particular findings can indicate that the planning and design of online learning environments for CPD should consider all the three categories of factors but most importantly the individual staff and ICT infrastructure related factors to ensure effective implementation of online learning for CPD.

### Perceived barriers to adopting online learning for CPD

**Fig. 7:**
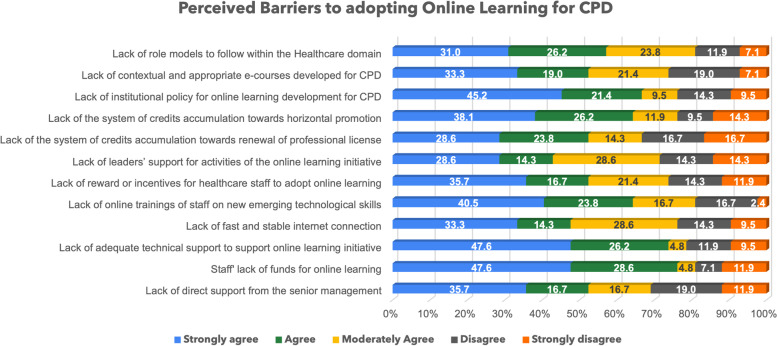
Perceived Barriers to adopting Online Learning for CPD

The data in Fig. 7 summarize the findings about the identified barriers as perceived by healthcare managers who participated in this study.

Based on these findings in Fig. 7, it is portrayed that all the proposed potential barriers to adopting online learning are considered to a larger extent by respondents in study although not at the same scale. More particularly.

- Lack of role models to follow within the healthcare domain.
- Lack of training on emerging digital skills and.
- Staff’s lack of funding to undertake CPD via online learning mode are reported as the key inhibitors that hinder the adoption of online learning for CPD.

All these three barriers were reported at (81%) degree of agreement respectively. Overall, all the barriers proposed in the survey questionnaire recorded more than (65%) degree of agreement by health managers who participated in this study.

**Fig. 8:**
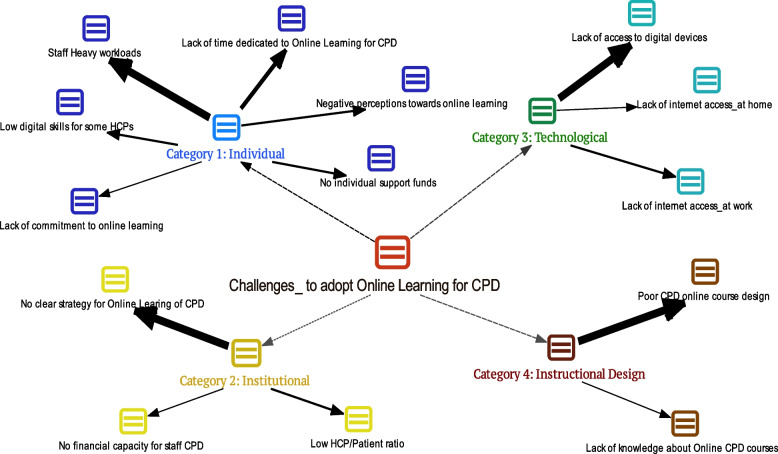
Reported challenges to adopting online learning for CPD. (Wider arrows indicate higher importance)

In this study, we also conducted interviews to get a deep understanding of the factors and challenges to the use of online learning systems for CPD. A summary of the findings from a thematic analysis of the interview data has been visualized and presented in Fig. 8 below. In this figure, the blue nodes represent a category of the reported individual challenges to adopting online learning for CPD while the yellow nodes portray the institutional level challenges. Accordingly, the technological related challenges are visualized in green nodes while the maroon red nodes represent the challenges related to the instructional CPD course design. The more the size of the allow from each category, the more the magnitude a particular challenge was discussed and emphasized by participants during the interviews.

The challenges to adopting online learning for CPD where clustered into 4 categories, namely (1) individual, (2) institutional, (3) technological and (4) instructional design as visualized in Fig. 8. The most critical challenges for online learning as reported by the healthcare managers are reported in this study are (1) *Staff heavy workloads*, (2) *No clear strategy for online learning of CPD*, (3) *Lack of access to digital devices* and (4) *Poor CPD online course design*. The individual category recorded six challenges while the institutional and technological categories recorded three challenges followed with Instructional design category with two challenges.

## Discussion

In this study, we explored healthcare managers’ perspectives concerning the importance of online learning for CPD delivery. The study also assessed the managers’ perception of the availability of digital infrastructure that supports online learning. Moreover, the study identified a number of enhancing factors and barriers to adopting online learning for CPD delivery from the perspective of healthcare managers. While the factors related to individual health professionals as well as ICT infrastructure are most important to consider, the institutional related factors should also not be ignored at all.

The findings also revealed that managers consider online learning as a strategy that can enhance CPD delivery. Managers who participated in this study reported online CPD as very important (90.5%) and rather important (9.5%). All managers (100%) consider online CPD as a strategy that can improve the knowledge, attitude, and practice of healthcare professionals. However, the majority of managers prefer a blended learning mode for CPD (90.5%). Regarding the status of digital infrastructure from managers’ perspective, the results indicated that wireless connection is excellent and good (85.7%). They also reported that mobile broadband is excellent and good (61.9%). Because of the small sample size, the difference of internet connection between rural and urban areas was not ascertained.

On the other hand, the study also revealed important barriers to implementing online CPD. The findings indicate that some health institutions have not started using online CPD, and the majority have not incorporated online CPD in institutions’ strategic plans. Most institutions (76.2%) also do not have a budget aligned with online CPD. In addition, managers reported barriers that hinder an effective implementation of online learning for CPD including the lack of training about emerging technologies related to online CPD, lack of role models, lack of funds, lack of fast and stable internet, and lack of adequate technical support. Furthermore, the analysis from the interviews done with managers of health institutions highlighted many barriers to the implementation of online CPD. They highlighted barriers like heavy staff workload that can result in lack of time for online CPD, anticipated lack of commitment to online CPD, lack of access to digital devices, lack of internet access at work or home, poorly designed online CPD courses, and lack of knowledge about online CPD courses.

The importance of online CPD aligns with previous studies and indicates that online learning is an important strategy for improving knowledge and skills. A study carried out by Feldacker et al. [9] found that online CPD is accepted in Sub-Saharan Africa and increases the opportunity of bringing expert knowledge and skills to healthcare professionals. In this study, managers reported factors that enhance online CPD programs as the need to accumulate credits for renewal of professional license and the need to earn credits for horizontal promotion. These two factors are vital in enhancing online learning adoption for CPD. Although these two motives are extrinsic, they similarly indicate that online CPD is a common interest for healthcare professionals and health institutions’ managers that could be built on to strengthen the online learning approach for CPD.

On the other hand, managers acknowledged the importance of online CPD, but they mostly prefer blended learning for CPD (online learning combined with face-to-face sessions). This attitude of managers confirms other study findings, which indicated superior importance of blended learning compared to approaches of pure online and only face to face. Studies report that pure online learning increases knowledge and skills, but blended learning affects knowledge, skills, attitude, and behaviors [35, 36]. Since knowledge and skills are not sufficient for the best service delivery, the willingness of healthcare managers to incorporate a blended learning mode for CPD rather than pure or only face-to-face learning would give more positive results to patients’ outcomes. Moreover, following the identified barriers, like low digital skills for some healthcare professionals, low access to digital services, unstable internet connectivity, and poor CPD online course designs, using face-to-face sessions to complement online learning would enhance the achievement of learning goals.

In addition, the findings from this study align with the previous study findings [2, 21]on the barriers to implementing online learning for CPD as inadequate online infrastructure, lack of adequate support on E-learning tools, low or no digital skills, poor internet connectivity, limited availability of electronic devices, and time constraints of healthcare professionals were also reported in this study. On the other hand, like this current study, these similar studies proposed strategies to overcome the challenges of implementing online CPD like adequate technical support, an online approach that is based on sound adult learning theories, institutional support to promote online CPD, increase in the availability of digital devices and a reliable internet connectivity [37–39].

Thus, health institution managers should capitalize on the importance they attribute to online CPD and avail necessary institutional support such as access to a reliable internet connection and digital devices (laptops and tablets) at the workplace. They should also consider staff workloads reduction, particularly at the health center level, time allocation for online CPD, and improve the design of online CPD courses.

Similarly, poor instructional and pedagogical design of online CPD courses is a critical challenge as revealed in this study. Online CPD training programs should be organized following sound instructional design principles such as goal-oriented, self-paced, and based on healthcare professionals’ learning needs. Accordingly, online CPD courses should also be designed based on real-life practices of healthcare professionals and the overall adult learning styles.

### Study limitations

The findings from this study are based on the analysis of surveys and interviews with healthcare managers. The mixed methods design which was used by concurrently triangulating quantitative and qualitative approaches for data collection and analysis may have produced some bias originating from interpreting two different data types. Also, the response rate to the survey is not highly satisfactory, which prevent the generalization of this study findings to other health sectors’ settings. While the participants were purposely included in the study based on their position regarding institutions’ plans regarding online learning, there might be over or under-reporting in their responses. Moreover, the sample size of 42 participants was rather small for carrying out statistical tests, even if they represent the majority of healthcare institutions in the country. Thus, the findings for this study cannot be generalized to the entire population and therefore, rigorous approaches like experimental research considering a big and representative sample size to understand the effectiveness of online CPD for healthcare professionals in Rwanda and the region is highly proposed.

Moreover, triangulation of the survey with the observation of online CPD practice would reveal more valid findings of online CPD for healthcare professionals. In addition, this study was limited to the perspectives of healthcare managers regarding online CPD; thus, online CPD studies from healthcare professionals’ perspectives could indicate the actual picture of practicing online CPD in Rwanda or the regional scale.

Although online and computer-based CPD delivery was the main focus for this present study, it cannot be ignored that there are still some CPD courses where healthcare professionals need in-person lectures and practical face-to-face workshops. This study was limited only on how technology can support online CPD delivery to enable HCPs acquire new professional skills which in turn may address the shortage in human resource for health (HRH). The fact that some computer-based CPDs do not seem to be applicable to all CPD activities and related intended learning outcomes, especially those requiring in-person and practical sessions, further research is recommended, to analyze the relevance and/or hindrances of a successful face-to-face CPD from healthcare managers consideration and focusing on low-resource settings with a high shortage in HRH.

## Conclusion

Healthcare managers highly consider the importance of online learning for CPD delivery. Based on the study findings, healthcare managers reported that healthcare professionals could acquire knowledge, skills, and ethical attitudes through CPD courses within a blended learning mode. This study also indicated that healthcare professionals might be motivated to use online learning systems for CPD because they can earn credits for renewing their professional licenses and providing high-quality healthcare services. Therefore, this positive consideration of online CPD can be based on improving the identified barriers of low digital skills for some healthcare professionals, poor access to digital services, unstable internet connectivity, and poor online CPD course designs.

Despite the expressed importance of online learning for CPD in low-resource settings such as health centers in rural areas in Rwanda, the traditional CPD courses that require in-person training sessions in the form of workshops, seminars or demonstrations to acquire hands-on practical skills are still recommended. Overall, these traditional face-to-face CPD courses are advocated for more particularly in the health centers where internet and related technologies are still scarce.

Afterwards, the ability for healthcare managers to address the barriers to adopting and using online learning for CPD as revealed in this study, such as low connectivity, lack of access to digital devices and related services, needs to be considered within the broader health systemic issues that currently confront the country in terms of resourcing and technological infrastructure. A strong partnership is therefore recommended to create this ability of healthcare managers to institutionalize the use of online learning for CPD by enabling co-creation and collaboration of all the managerial revels of the pyramidal structure of the Rwandan health system and other potential external health partners.

## Data Availability

The datasets generated and/or analyzed during the current study are not publicly available because we do not have ethical approval for making these raw data available for sharing. However, they can be obtained from the corresponding author on reasonable request.

## References

[1] Ganzamungu Z, Ross AJ, Dumsani MG, MacGregor RG. A review on the contributions of NGOs in addressing the shortage of healthcare professionals in rural South Africa. Cogent Soc Sci. 2019;5(1):1–18.

[2] Ngenzi JL, Scott RE, Mars M (2021). Information and communication technology to enhance continuing professional development (CPD) and continuing medical education (CME) for Rwanda: a scoping review of reviews. BMC Med Educ.

[3] WHO. Global health observatory data repository. 2018. https://apps.who.int/gho/data/node.main.HWFGRP_0020?lang=en. Accessed 18 June 2021.

[4] RAHPC. Continuing Professional Development (CPD) Policy for Health Professional Councils in Rwanda. Kigali, Rwanda; 2013.

[5] Filipe HP, Silva ED, Stulting AA, Golnik KC (2014). Continuing professional development: best practices. Middle East Afr J Ophthalmol.

[6] McCutcheon K, O’Halloran P, Lohan M (2018). Online learning versus blended learning of clinical supervisee skills with pre-registration nursing students: a randomised controlled trial. Int J Nurs Stud.

[7] Gould D, Papadopoulos I, Kelly D (2014). Tutors’ opinions of suitability of online learning programmes in continuing professional development for midwives. Nurse Educ Today.

[8] Reeves S, Fletcher S, McLoughlin C, Yim A, Patel KD (2017). Interprofessional online learning for primary healthcare: findings from a scoping review. BMJ Open.

[9] Feldacker C, Jacob S, Chung MH, Nartker A, Kim HN (2017). Experiences and perceptions of online continuing professional development among clinicians in sub-saharan Africa. Hum Resour Health.

[10] Chime JK, Munyati P, Katepa-Bwalya M, Musumali M, Mweetwa B, Kagulura S (2016). Using e-Learning for skills transfer, motivation and retention of health workers in Zambia. Med J Zambia.

[11] Barteit S, Jahn A, Bowa A, Lüders S, Malunga G, Marimo C (2018). How self-directed e-learning contributes to training for medical licentiate practitioners in Zambia: evaluation of the pilot phase of a mixed-methods study. JMIR Med Educ.

[12] García Vazquez A, Verde JM, Dal Mas F, Palermo M, Cobianchi L, Marescaux J (2020). Image-guided surgical e-learning in the post-COVID-19 pandemic era: what is next?. J Laparoendosc Adv Surg Tech.

[13] Regmi K, Jones L (2020). A systematic review of the factors–enablers and barriers–affecting e-learning in health sciences education. BMC Med Educ.

[14] Berndt A, Murray CM, Kennedy K, Stanley MJ, Gilbert-Hunt S (2017). Effectiveness of distance learning strategies for continuing professional development (CPD) for rural allied health practitioners: a systematic review. BMC Med Educ.

[15] Colaceci S, Giusti A, Chapin EM, Bettinelli ME, De Angelis A, Zambri F (2017). E-learning to improve healthcare professionals’ attitudes and practices on breastfeeding. Breastfeed Med.

[16] Harerimana A, Mtshali NG, Ewing H, Maniriho F, Kyamusoke E, Mukankaka A (2016). E-learning in nursing education in Rwanda: benefits and challenges. Explor Participants’ Perceptives.

[17] Barteit S, Guzek D, Jahn A, Bärnighausen T, Jorge MM, Neuhann F (2020). Evaluation of e-learning for medical education in low-and middle-income countries: a systematic review. Comput Educ.

[18] Gawugah JN, Jadva-Patel H, Jackson MT (2011). The uptake of continuing professional development (CPD) by ghanaian radiographers. Radiography.

[19] Ministry of Health. Health Labor Market Analysis Report. Kigali; 2019.

[20] Sandars J, Langlois M, Waterman H (2007). Online collaborative learning for healthcare continuing professional development: a cross-case analysis of three case studies. Med Teach.

[21] O’Doherty D, Dromey M, Lougheed J, Hannigan A, Last J, McGrath D (2018). Barriers and solutions to online learning in medical education–an integrative review. BMC Med Educ.

[22] Kyalo IW, Hopkins S (2013). Exploring the acceptability of online learning for continuous Professional Development at Kenya Medical Training Colleges. Electron J E-learning.

[23] Weber DL, Cubaka VK, Kallestrup P, Reventlow S, Chriver M (2020). Rwandan primary healthcare providers’ perception of their capability in the diagnostic practice. Afr J Prim Health Care Fam Med.

[24] Dunleavy K, Chevan J, Sander AP, Gasherebuka JD, Mann M (2018). Application of a contextual instructional framework in a continuing professional development training program for physiotherapists in Rwanda. Disabil Rehabil.

[25] Rusatira JC, Tomaszewski B, Dusabejambo V, Ndayiragije V, Gonsalves S, Sawant A (2016). Enabling access to medical and health education in Rwanda using mobile technology: needs assessment for the development of mobile medical educator apps. JMIR Med Educ.

[26] Harerimana A, Mtshali NG (2018). Implementing e-learning in resource-constrained nursing education institutions in Rwanda. Res Rev J Nurs Health Sci.

[27] Gardner P, Slater H, Jordan JE, Fary RE, Chua J, Briggs AM (2016). Physiotherapy students’ perspectives of online e-learning for interdisciplinary management of chronic health conditions: a qualitative study. BMC Med Educ.

[28] Creswell JW (2014). Research design: qualitative, quantitative, and mixed methods approaches.

[29] Morgan DL (2014). Integrating qualitative and quantitative methods: a pragmatic Approach.

[30] Denscombe M (2010). The Good Research Guide: for small-scale social research.

[31] Strauss A, Corbin J (1990). Basics of qualitative research.

[32] Mero-Jaffe I (2011). “Is that what I said?” Interview transcript approval by participants: an aspect of ethics in qualitative research. Int J Qual Methods.

[33] Corbin J (2008). Basics of qualitative research: techniques and procedures for developing grounded theory.

[34] Cohen L, Manion L, Morrison K (2013). Research methods in education.

[35] Rohwer A, Motaze NV, Rehfuess E, Young T (2017). E-learning of evidence-based health care (EBHC) to increase EBHC competencies in healthcare professionals: a systematic review. Campbell Syst Reviews.

[36] Liu Q, Peng W, Zhang F, Hu R, Li Y, Yan W. The effectiveness of blended learning in health professions: Systematic review and meta-analysis. J Med Internet Res. 2016;18(1):e4807. 10.2196/jmir.4807 PMC4717286 26729058

[37] Ngenzi JL, Scott RE, Mars M. Information and communication technology to enhance continuing professional development (CPD) and continuing medical education (CME) for Rwanda: a scoping review of reviews. BMC Med Educ. 2021;21:245. 10.1186/s12909-021-02607-w PMC8081763 33926419

[38] O’Doherty D, Dromey M, Lougheed J, Hannigan A, Last J, McGrath D. Barriers and solutions to online learning in medical education - an integrative review. BMC Med Educ. 2018;18:130. 10.1186/s12909-018-1240-0 PMC5992716 29880045

[39] Gawugah JNK, Jadva-Patel H, Jackson MT. The uptake of Continuing Professional Development (CPD) by Ghanaian radiographers. Radiography. 2011;17:332–44.

